# The College Conferences and Subsequent Gains

**Published:** 1918-06-01

**Authors:** 


### THE MATRONS' AND SISTERS' DEPARTMENT.

### XIV.—THE COLLEGE CONFERENCES AND SUBSEQUENT GAINS.

THE coming Conference of the College of Nursing, fixed to take place on June 6 and 7, while matrons and nurses are in town for the annual meeting, marks a new departure in nursing conferences. The common defect of such meetings has been too often aimlessness. Excellent contributions have often been made by persons well acquainted with their subject to the vexed problems of training, pay, and employment; but none of those present have ever had power to alter existing conditions. The resolutions passed, the recommendations agreed to, have passed away with the enthusiasm of the hour. The motive power has been wanting. From henceforth, in conferences held by members of the College, this sense of emptiness and wasted breath should disappear. To those who bring up the familiar complaint, "Cui bono?" the answer is ready. These nurses assembled for discussion hold enormous power in their hands. Some of the speakers may be members of the College Council, some, like Miss Gullan, may be actively co-operating in union with those who influence the policy of the College, some may be distinguished members of the College not yet in office. But, whatever the speaker, the resolutions carried, the recommendations agreed to, will be considered by a body of nurses who hold the power in their hands to return members of the Council pledged to carry through these reforms. What is agreed upon in conference is likely to become a "plank" in the College policy. There is at last some definite hope that the united voices of the nursing world will come to influence the event, and that matters will be amended where they are conspicuously at fault. This, then, is "the good of it"—that the nurses who attend will do so not to talk or merely to hear each other talk, but to come to a decision as to what things need to be done. They will give up other engagements and travel great distances at great expense for a purpose—to hear all sides of many vexed questions placed before them, so that they may reach an impartial judgment. Once members have come to form such judgments, it follows that, members of the Council will be elected more and more with a view to carrying things through which are seen to be just and helpful for nurses generally. In short, the foundations of a wise and coherent policy have to be laid in conference.

The defect, generally acknowledged, in the constitution of the Royal British Nurses' Association is that, it does not encourage the independent exercise of judgment on the part of its members. On its Council and Executive Committee one-third are doctors, two-thirds matrons and nurses. But it is perhaps inevitable that the doctors have come to hold a preponderating position in its affairs. Of the three honorary officers, moreover, two, the medical honorary secretary and the honorary treasurer, are men. It would be ridiculous to assert that the doctors connected with this Association have used the powers entrusted to them by its constitution otherwise than unselfishly for the common good. They have sacrificed much time and energy to its affairs; but their very efficiency has tended to discourage initiative among the women members. Busy women are strongly tempted to allow someone else to manage their affairs, and in a society this attitude leads in the direction of apathy. Men and women work very well together on committees connected with philanthropic and municipal affairs. But when it comes to professional interests, when all the women belong to one profession and all the men to another, when, moreover, the one profession is linked in closest subordination with the other, then the situation may become extremely difficult. Either an attitude of friction and revolt must be engendered among a majority which feels itself to be dominated, or a peaceful *entente* is established, the doctors performing with good will many kindly offices for the good of nurses, as they see it, and the matrons and nurse members sitting placidly by while their affairs are settled, observing to each other with a tinge of gentle irony, how kind it is of the doctors to take so much trouble for them.

We are convinced that the only way to solve the numerous pressing problems which afflict the nursing world is for the onus of reform to be thrown on the matrons and nurses themselves. They will not master these questions unless they are left free to tackle them in their own way; unless they are convinced, moreover, that no one else has the ability to do it for them. But the burden of making these momentous decisions ought not to rest on the shoulders of a few only. The whole body of nurses ought to study these questions, that they may take a wide view and be ready to support their leaders. In conference and private discussions members of the nursing profession can brush all crude impressions out of their heads. They have yet to learn the difference between short-cut reforms, which do but create new difficulties, and statesmanlike measures likely to endure. In this way can the College be built on a broad and sure foundation which will endure.

Previous articles Dec. 8, 15, 22, Jan. 5, 12, 26, Feb. 2, March 2, 9, 23, 30, April 13, and May 11, p. 123.

